# Epitope mapping of anti-mouse podoplanin monoclonal antibody PMab-1

**DOI:** 10.1016/j.bbrep.2018.07.002

**Published:** 2018-07-09

**Authors:** Shinji Yamada, Shunsuke Itai, Mika K. Kaneko, Satoru Konnai, Yukinari Kato

**Affiliations:** a Department of Antibody Drug Development, Tohoku University Graduate School of Medicine, 2-1 Seiryo-machi, Aoba-ku, Sendai, Miyagi 980-8575, Japan; b Department of Disease Control, Faculty of Veterinary Medicine, Hokkaido University, Sapporo, Hokkaido 060-0818, Japan; c New Industry Creation Hatchery Center, Tohoku University, 2-1, Seiryo-machi, Aoba-ku, Sendai, Miyagi 980-8575, Japan

**Keywords:** PDPN, podoplanin, PLAG, platelet aggregation-stimulating, mAb, monoclonal antibody, ELISA, enzyme-linked immunosorbent assay, PBS, phosphate-buffered saline, DAB, 3,3-diaminobenzidine tetrahydrochloride, Podoplanin, PDPN, PMab-1, Epitope mapping

## Abstract

Mouse podoplanin (mPDPN) is a type I transmembrane sialoglycoprotein, which is expressed on lymphatic endothelial cells, podocytes of the kidney, and type I alveolar cells of the lung. mPDPN is known as a platelet aggregation-inducing factor and possesses four platelet aggregation-stimulating (PLAG) domains: PLAG1, PLAG2, and PLAG3 in the N-terminus and PLAG4 in the middle of the mPDPN protein. mPDPN overexpression in cancers has been reportedly associated with hematogenous metastasis through interaction with the C-type lectin-like receptor 2 of platelets. We previously reported a rat anti-mPDPN monoclonal antibody clone PMab-1, which was developed by immunizing the PLAG2 and PLAG3 domains of mPDPN. PMab-1 is very useful in flow cytometry, western blot, and immunohistochemical analyses to detect both normal cells and cancers. However, the binding epitope of PMab-1 remains to be clarified. In the present study, flow cytometry, enzyme-linked immunosorbent assay, and immunohistochemical analyses were utilized to investigate the epitope of PMab-1. The results revealed that the critical epitope of PMab-1 is Asp39 and Met41 of mPDPN. These findings can be applied to the production of more functional anti-mPDPN monoclonal antibodies.

## Highlights

• Sensitive and specific anti-mPDPN mAb, PMab-1 was previously established.
• PMab-1 is useful in flow cytometry, Western blot, and immunohistochemical analyses.
• The critical epitope of PMab-1 was determined to be Asp39 and Met41 of mPDPN.
• PMab-1 reaction was neutralized by epitope peptide of mPDPN.

## 1 Introduction

Podoplanin (PDPN/T1alpha/gp38/Aggrus) is expressed in many normal tissues, such as renal podocytes, lymphatic endothelial cells of many tissues, and pulmonary type I alveolar cells [1], [2], [3], [4]. Several anti-mouse PDPN (mPDPN) monoclonal antibodies (mAbs), such as clone 8.1.1 or clone PMab-1, have been used in many studies [5]. However, clone 8.1.1 is produced using hamsters, and clone PMab-1 is produced using rats because it is difficult to develop anti-mPDPN mAbs using mice. Recently, we developed a rat–mouse chimeric antibody, mPMab-1 of mouse IgG_2a_, which was derived from a rat PMab-1 mAb [6]. Immunohistochemical analysis showed that mPMab-1 detects podocytes of the kidney, lymphatic endothelial cells of the colon, and type I alveolar cells of the lung. Importantly, mPMab-1 was shown to be more sensitive than original PMab-1.

mPDPN possesses four platelet aggregation-stimulating (PLAG) domains: PLAG1, PLAG2, and PLAG3 in the N-terminus [1] and PLAG4 in the middle of the mPDPN protein [7]. In a previous study, PMab-1 mAb was produced against the platelet aggregation-stimulating (PLAG) domain of mPDPN [5]; therefore, PMab-1 neutralizes the interaction between mPDPN and the C-type lectin-like receptor 2 [8], [9], [10]. The administration of PMab-1 was found to reduce lymphangiogenesis in corneal suture and ear-wound healing models [11]. PMab-1 also suppressed the infiltration of thioglycollate-induced macrophages at the site of wound healing. Furthermore, the administration of PMab-1 lead to a significant suppression of the rejection reaction in a corneal transplantation model, suggesting that mPDPN is a novel therapeutic target for suppressing lymphangiogenesis and inflammation.

In the present study, we determined the binding epitope of PMab-1 to mPDPN using flow cytometry, enzyme-linked immunosorbent assay (ELISA), and immunohistochemical analyses.

## 2 Materials and methods

### 2.1 Cell line

Chinese hamster ovary (CHO)-K1 cell line was purchased from the American Type Culture Collection (Manassas, VA, USA). The mPDPN mutation plasmids were transfected into CHO-K1 cells using Lipofectamine LTX (Thermo Fisher Scientific Inc., Waltham, MA, USA). Transiently transfected cells were cultured in RPMI 1640 medium (Nacalai Tesque, Inc., Kyoto, Japan) supplemented with 10% heat-inactivated fetal bovine serum (Thermo Fisher Scientific Inc.), 100 units/ml of penicillin, 100 μg/ml of streptomycin, and 25 μg/ml of amphotericin B (Nacalai Tesque, Inc.) at 37 °C in a humidified atmosphere of 5% CO_2_ and 95% air.

### 2.2 Production of mPDPN point mutants

The cDNA of mPDPN was subcloned into a pcDNA3 vector (Thermo Fisher Scientific Inc.) [2]. Substitutions of amino acids to alanine in the mPDPN sequence were performed using a QuikChange Lightning Site-Directed Mutagenesis Kit (Agilent Technologies Inc., Santa Clara, CA, USA).

### 2.3 Flow cytometry

Cells were harvested after brief exposure to 0.25% trypsin/1 mM ethylenediaminetetraacetic acid (Nacalai Tesque, Inc.). After washing with 0.1% bovine serum albumin in PBS, the cells were treated with PMab-1 for 30 min at 4 °C, followed by treatment with Alexa Fluor 488-conjugated anti-rat IgG (1:1000; Cell Signaling Technology, Inc., Danvers, MA). Fluorescence data were acquired using the Cell Analyzer EC800 (Sony Corp., Tokyo, Japan).

### 2.4 ELISA

Synthesized mPDPN peptides using PEPScreen (Sigma-Aldrich Corp., St. Louis, MO) were immobilized on Nunc Maxisorp 96-well immunoplates (Thermo Fisher Scientific Inc.) at 10 μg/ml for 30 min at 37 °C. After blocking with SuperBlock T20 (PBS) Blocking Buffer (Thermo Fisher Scientific Inc.), the plates were incubated with purified PMab-1 (10 μg/ml), followed by a 1:2000 dilution of peroxidase-conjugated anti-rat IgG (Agilent Technologies Inc.). The enzymatic reaction was performed using 1-Step Ultra TMB-ELISA (Thermo Fisher Scientific Inc.). Optical density was measured at 655 nm using an iMark microplate reader (Bio-Rad Laboratories, Inc., Berkeley, CA). These reactions were performed at 37 °C with a total sample volume of 50–100 μl.

### 2.5 Immunohistochemical analyses

Histological sections (4-μm thick) of mouse tissues were directly autoclaved in citrate buffer (pH 6.0; Nichirei Biosciences, Inc., Tokyo, Japan) for 20 min. After blocking with SuperBlock T20 (PBS) Blocking Buffer (Thermo Fisher Scientific Inc.), sections were incubated with mPMab-1 (1 μg/ml) or mPMab-1 (1 μg/ml) plus peptides (5 μg/ml) for 1 h at room temperature and treated using an Envision + kit (Agilent Technologies Inc.) for 30 min. Color was developed using 3,3-diaminobenzidine tetrahydrochloride (DAB; Agilent Technologies Inc.) for 2 min. Sections were counterstained with hematoxylin (FUJIFILM Wako Pure Chemical Corporation, Osaka, Japan).

## 3 Results and discussion

In a previous study, we developed a rat anti-mPDPN mAb PMab-1 by immunizing the PLAG domain of mPDPN [5]. We further produced a rat–mouse chimeric antibody, mPMab-1 of mouse IgG_2a_, which was derived from a rat PMab-1 mAb [6]. Immunohistochemical analysis showed that both PMab-1 and mPMab-1 are capable of detecting podocytes of the kidney, lymphatic endothelial cells of the colon, and type I alveolar cells of the lung. Interestingly, mPMab-1 was shown to be more sensitive than original PMab-1 [6] probably because a high-sensitivity immunohistochemical kit can be used for mouse IgG. In the present study, we produced point mutants of mPDPN (proteins and synthesized peptides) and investigated the critical epitope of PMab-1 for mPDPN detection.

**Fig. 1:**
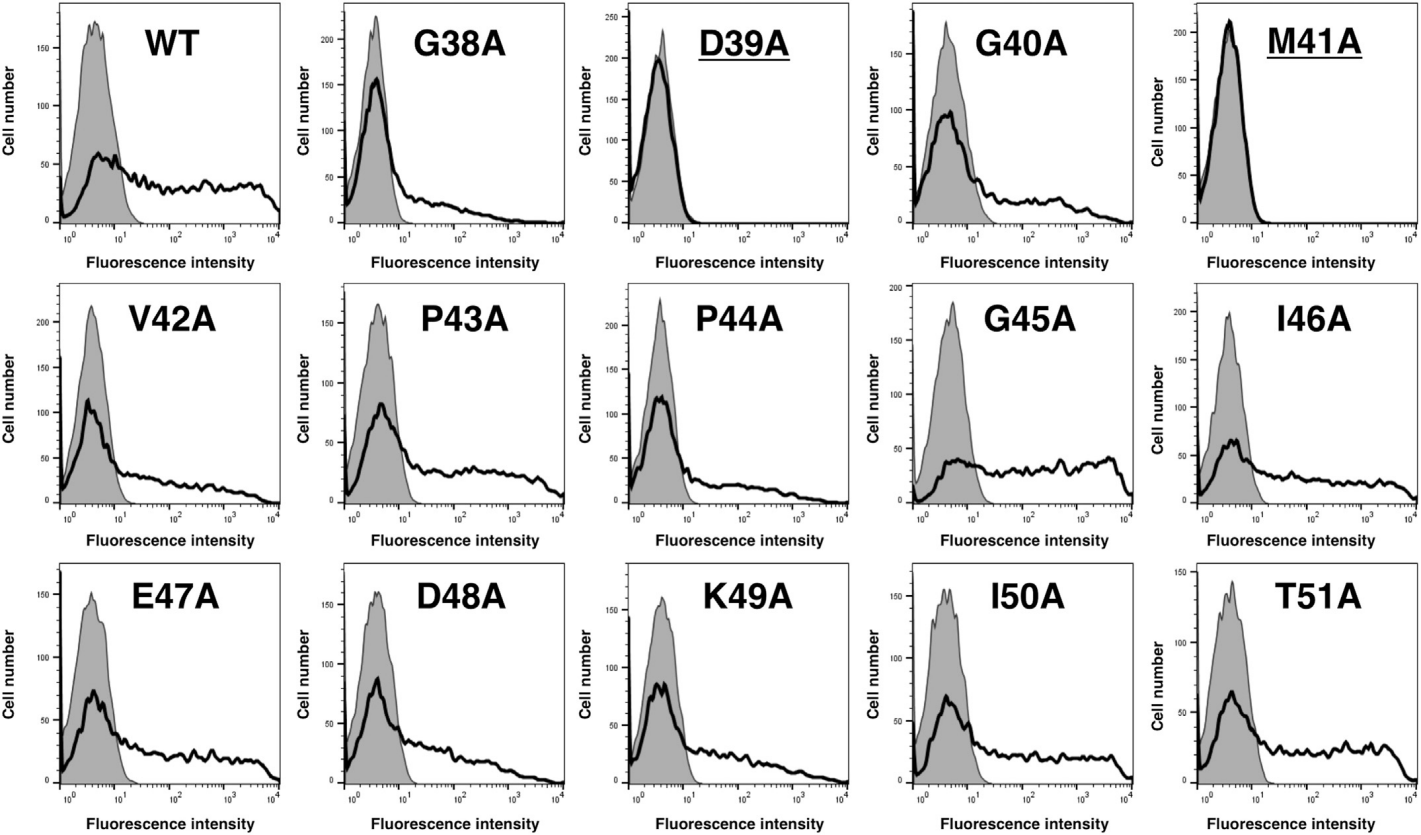
**Epitope mapping of PMab-1 using point mutants of mPDPN.** Point mutants of mPDPN were analyzed using flow cytometry. Point mutants were expressed on CHO-K1 cells and then incubated with PMab-1 (2 μg/ml) or buffer control for 30 min at 4 °C, followed by treatment with corresponding secondary antibodies.

Because PMab-1 was developed by immunizing rats with amino acids 38–51 of mPDPN, we produced a series of point mutants of mPDPN using a QuikChange Lightning Site-Directed Mutagenesis Kit. As shown in Fig. 1, PMab-1 reacted with G38A, G40A, V42A, P43A, P44A, G45A, I46A, E47A, D48A, K49A, I50A, and T51A in flow cytometry. In contrast, it did not react with D39A and M41A, indicating that Asp39 and Met41 of mPDPN are critical for PMab-1 recognition.

**Fig. 2:**
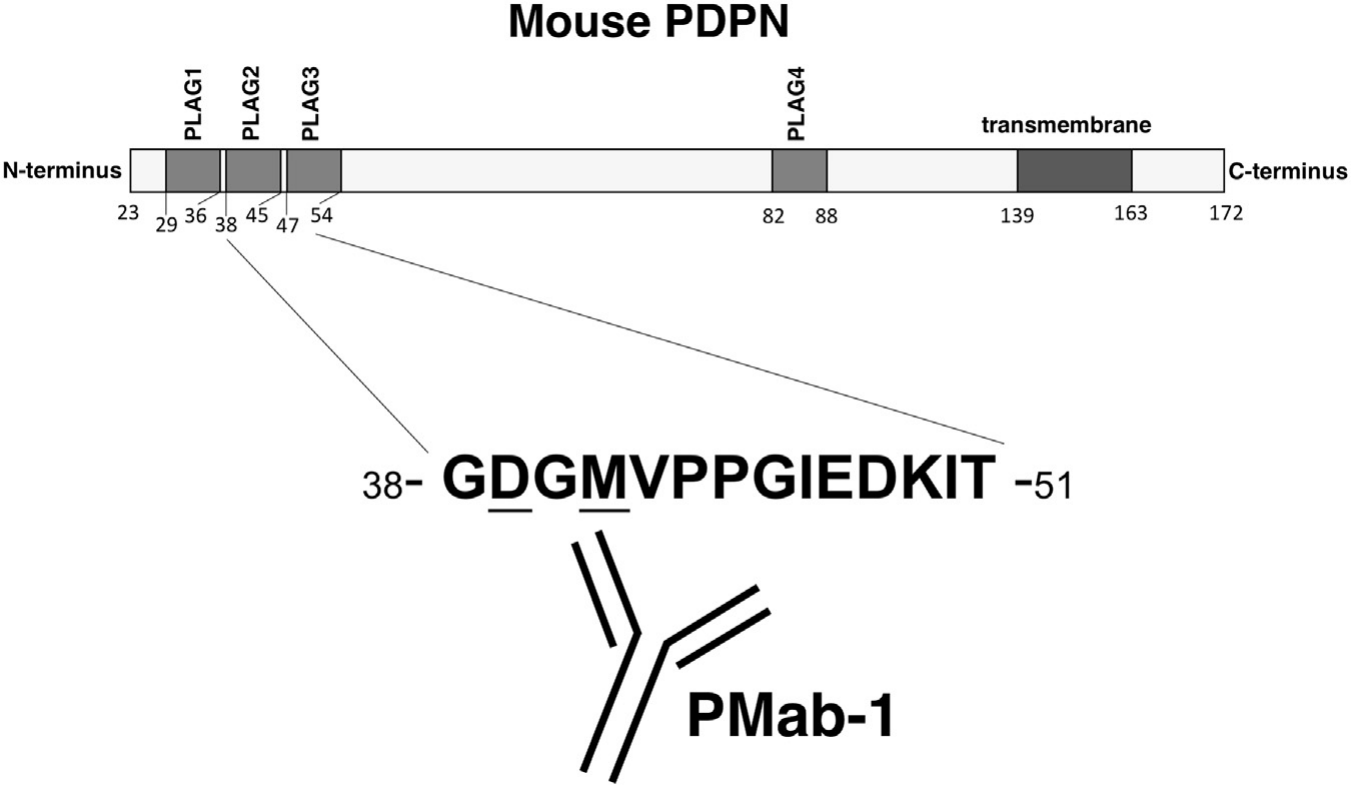
**Illustration of mPDPN and epitope of PMab-1.** mPDPN possesses four PLAG domains. PMab-1 was produced by immunizing PLAG2 and PLAG3 domains. Asp39 and Met41 are critical amino acids for PMab-1 recognition to mPDPN.

Next, we synthesized a series of point mutants of mPDPN peptides from the 38th to the 51st amino acid (Supplementary Table 1). Using ELISA, PMab-1 detected G38A, G40A, V42A, P43A, P44A, G45A, I46A, E47A, D48A, K49A, I50A, and T51A. In contrast, it did not recognize D39A and M41A, confirming the result from the flow cytometric analysis (Fig. 2).

**Fig. 3:**
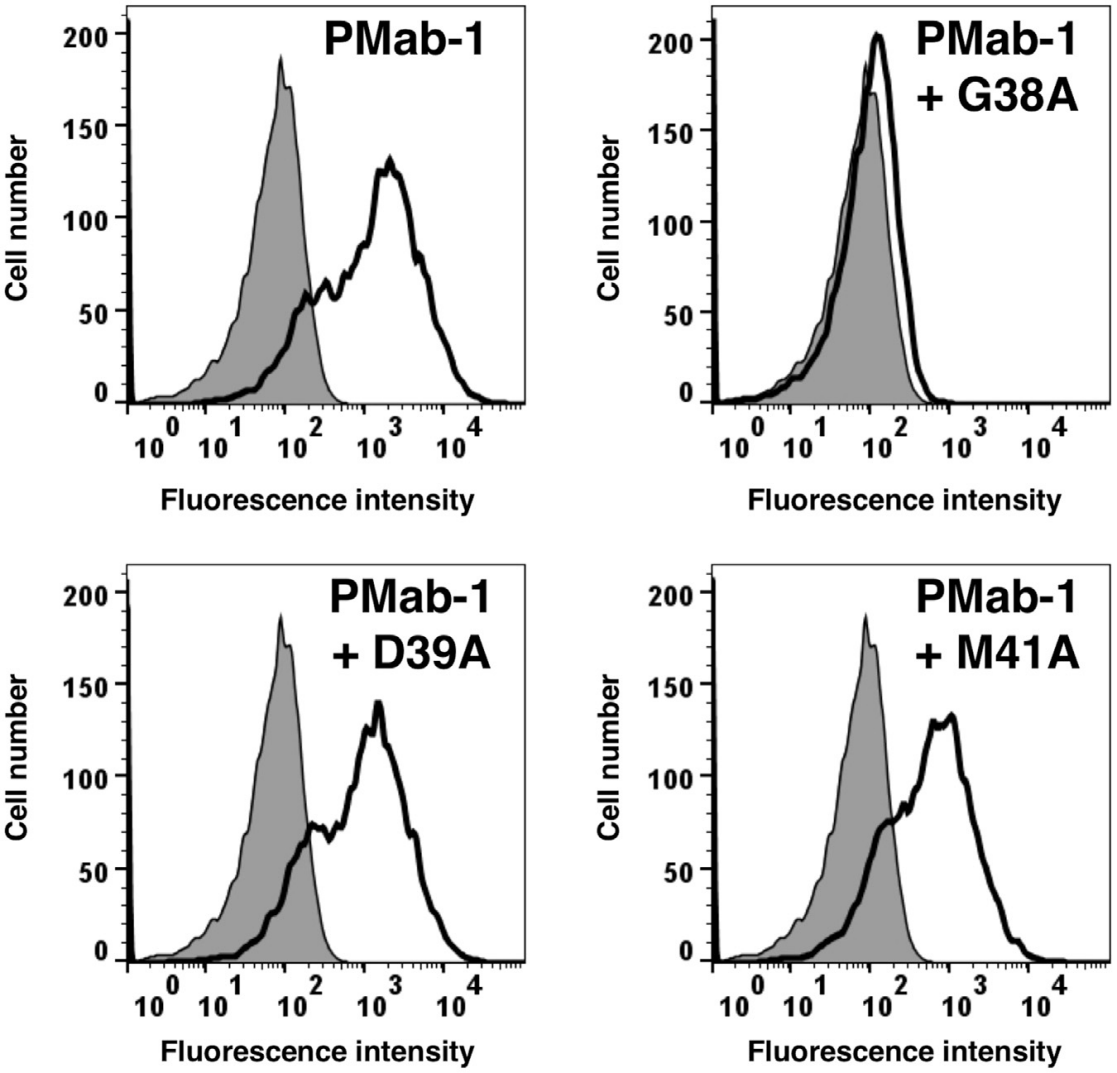
**Flow cytometry using PMab-1 and point mutants of mPDPN.** PMab-1 (1 μg/ml) or PMab-1 (1 μg/ml) plus peptides (G38A, D39A, and M41A; 10 μg/ml) were reacted with CHO/mPDPN cells for 30 min at 4 °C, followed by the addition of secondary antibodies.

We performed a blocking assay using flow cytometry. PMab-1 reacted with the CHO/mPDPN cell line (Fig. 3). This reaction was completely neutralized by G38A. In contrast, D39A and M41A did not block the reaction of PMab-1 with CHO/mPDPN, indicating that Asp39 and Met41 of mPDPN are critical for PMab-1 detection.

**Fig. 4:**
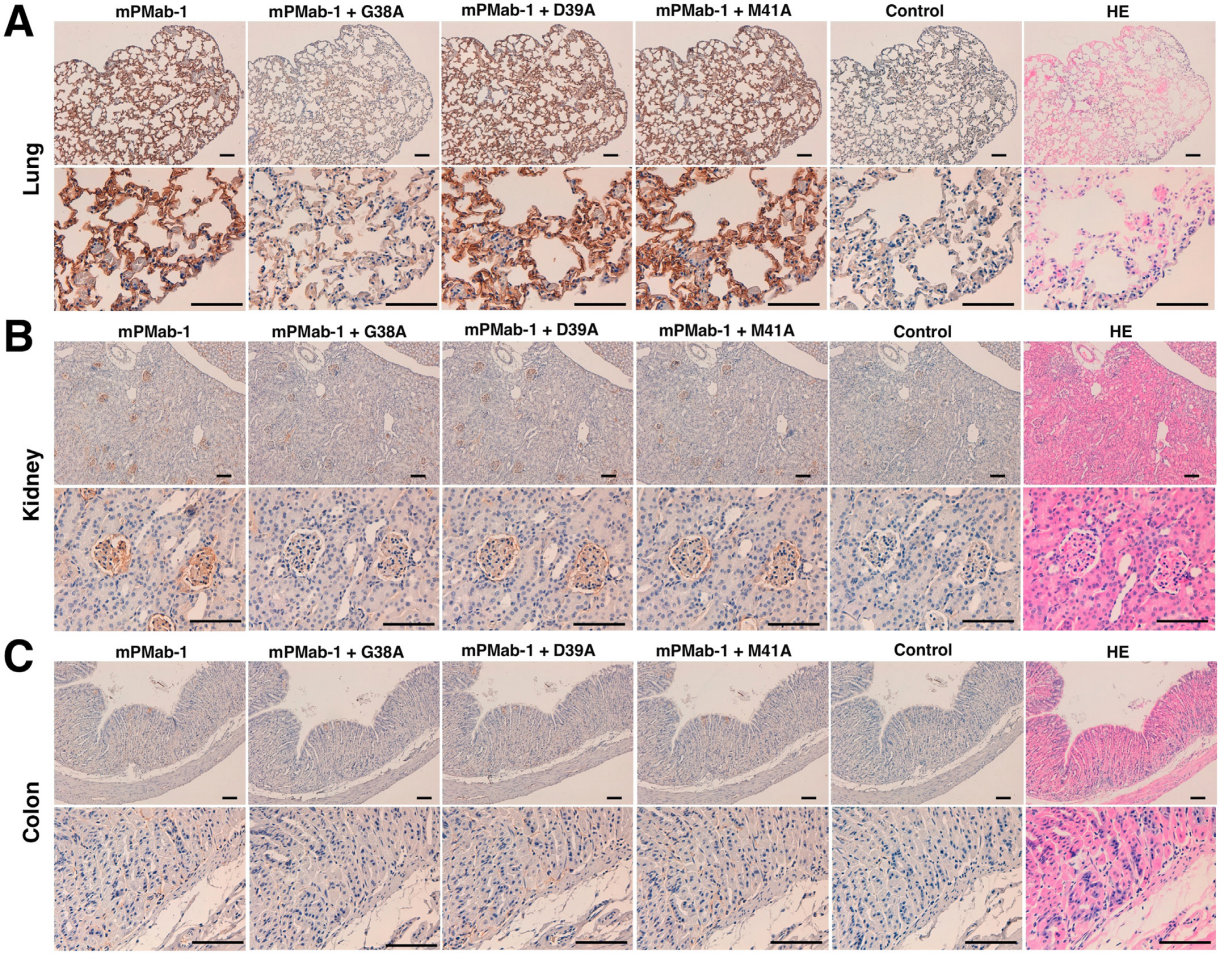
**Immunohistochemical analyses.** Histological sections of the lung (A), kidney (B), and colon (C) were directly autoclaved in citrate buffer for 20 min. After blocking with SuperBlock T20 (PBS) Blocking Buffer, sections were incubated with mPMab-1 (1 μg/ml) or mPMab-1 (1 μg/ml) plus peptides (5 μg/ml), followed by treatment with an Envision+ kit. HE, hematoxylin and eosin. Scale bar, 100 µm.

**Fig. S1:**
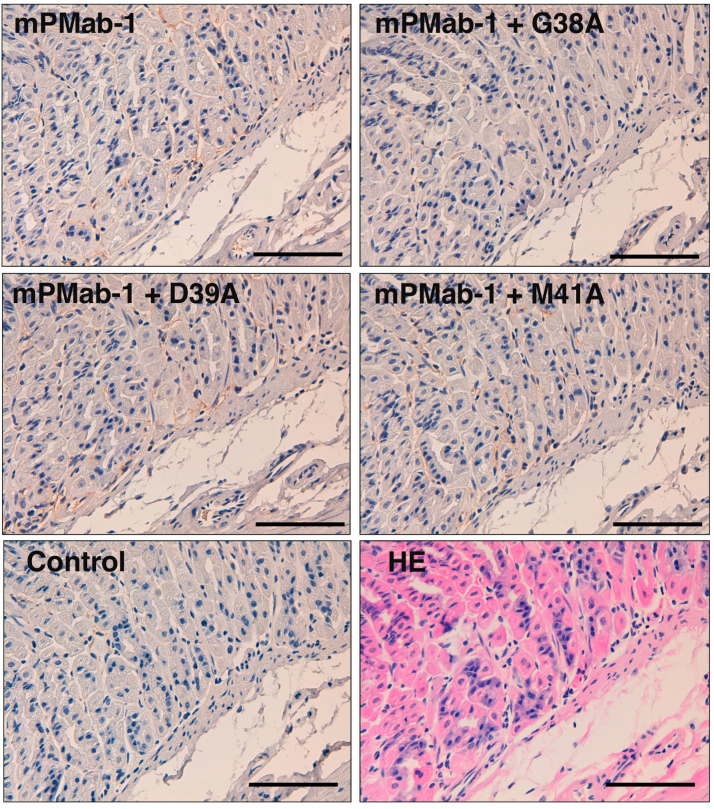
**Immunohistochemical analyses.** Histological sections of colon tissues were directly autoclaved in citrate buffer for 20 min. After blocking with SuperBlock T20 (PBS) Blocking Buffer, sections were incubated with mPMab-1 (1 μg/ml) or mPMab-1 (1 μg/ml) plus peptides (5 μg/ml), followed by treatment with an Envision+ kit. HE, hematoxylin and eosin. Scale bar, 100 µm. These pictures are magnification of Fig. 4C.

We further performed a blocking assay using immunohistochemistry. A rat–mouse chimeric mAb mPMab-1 reacted with type I alveolar cells (Fig. 4A), renal podocytes (Fig. 4B), and lymphatic endothelial cells of the colon (Fig. 4C and Supplementary Figure 1). These reactions were completely or partially neutralized by G38A. In contrast, D39A and M41A did not block these reactions of mPMab-1 with mouse tissues, indicating that Asp39 and Met41 of mPDPN are critical for PMab-1 detection.

Taken together, the critical epitope of PMab-1 is Asp39 and Met41 of mPDPN. These findings can be applied to the production of more functional anti-mPDPN mAbs.
