# Supplementary material for: Epitope mapping of anti-mouse podoplanin monoclonal antibody PMab-1
Source: Biochem Biophys Rep. 2018 Jul 9;15:52–6. doi: 10.1016/j.bbrep.2018.07.002 (PMC6039309; doi:10.1016/j.bbrep.2018.07.002)
Supplement: Supplementary file 2 — Supplementary material [file mmc2.doc]

Supplementary Table 1. Determination of PMab-1 Epitope by ELISA.

| *Mutation* | *Sequence* | *PMab-1* |
| --- | --- | --- |
|
| G38A | ADGMVPPGIEDKIT | ＋＋＋ |
| D39A | GAGMVPPGIEDKIT | − |
| G40A | GDAMVPPGIEDKIT | ＋＋ |
| M41A | GDGAVPPGIEDKIT | − |
| V42A | GDGMAPPGIEDKIT | ＋＋ |
| P43A | GDGMVAPGIEDKIT | ＋＋ |
| P44A | GDGMVPAGIEDKIT | ＋＋ |
| G45A | GDGMVPPAIEDKIT | ＋＋＋ |
| I46A | GDGMVPPGAEDKIT | ＋＋＋ |
| E47A | GDGMVPPGIADKIT | ＋＋＋ |
| D48A | GDGMVPPGIEAKIT | ＋＋＋ |
| K49A | GDGMVPPGIEDAIT | ＋ |
| I50A | GDGMVPPGIEDKAT | ＋＋＋ |
| T51A | GDGMVPPGIEDKIA | ＋＋＋ |

＋＋＋, OD655≧1.0; ＋＋, 0.6 ≦OD655＜1.0;
＋, 0.1≦OD655＜0.6; ―, OD655＜0.1.
